# PAXX Participates in Base Excision Repair via Interacting with Pol β and Contributes to TMZ Resistance in Glioma Cells

**DOI:** 10.1007/s12031-018-1157-4

**Published:** 2018-09-20

**Authors:** Ben Yang, Xueqi Fu, Jilong Hao, Jing Sun, Zongzhu Li, Haisong Li, Haiyang Xu

**Affiliations:** 1 Ophthalmologic Department, China-Japan Union Hospital of Jilin University, Changchun, China; 2 Edmond H. Fischer Signal Transduction Laboratory, School of Life Sciences, Jilin University, Changchun, China; 3 Ophthalmologic Department, First Hospital of Jilin University, Changchun, China; 4 Department of Biochemistry and Molecular Medicine, The George Washington University School of Medicine and Health Sciences, Washington, DC USA; 5 Department of Oncological Neurosurgery, First Hospital of Jilin University, Changchun, 130021 China

**Keywords:** PAXX, Non-homologous end joining, Temozolomide, Glioma

## Abstract

Non-homologous end joining (NHEJ) is one of the major DNA repair pathway in mammalian cell that can ligate a variety of DNA ends. However, how does all NHEJ factors communicate and organize together to achieve the final repair is still not clear. PAralog of XRCC4 and XLF (PAXX) was a new factor identified recently that play an important role in NHEJ. PAXX contributes to efficient NHEJ by interacting with Ku, which is a NHEJ key factor, and PAXX deficiency cause sensitivity to DNA double-strand break repair (DSBR). We observed that PAXX-deficient cells showed slight increase of homologous recombination (HR, which is another major DSBR repair pathways in mammalian cells). More importantly, we found that PAXX contributes to base excision repair pathway via interaction of polymerase beta (pol β). Temozolomide (TMZ) is one of the standard chemotherapies widely applied in glioblastoma. However, TMZ resistance and lack of potent chemotherapy agents can substitute TMZ. We observed that PAXX deficiency cause more sensitivity to TMZ-resistant glioma cells. In conclusion, the PAXX contributes to a variety of DNA repair pathways and TMZ resistance. Therefore, inhibition of PAXX may provide a promising way to overcome TMZ resistance and improve TMZ therapeutic effects in glioma treatment.

## Introduction

DNA double-strand breaks (DSBs), which happened commonly in human cells, are extremely toxic and inevitable that one DSB can induce cell death (Rassool 2003; Scully and Xie 2005; Seluanov et al. 2010). Un-repaired DSB can also result in mutations, chromosome translocations, and loss of genomic information (Burma et al. 2006; Chang et al. 2016). In order to maintain genome integrity, mammalian cells incorporated two major DSB repair pathways: HR and NHEJ (Deriano and Roth 2013). HR requires template to repair DSB accurately and is majorly active in S and G2 phases of cell cycle where sister chromatids are available (Zaunbrecher et al. 2008; Gerelchuluun et al. 2015). The step that is unique to HR pathway is DNA end resection, which can create a long 3′-overhang to find and invade in the sister chromatid (Cejka 2015).

Whereas NHEJ does not need template to join DSB (Kim et al. 2005; Gerelchuluun et al. 2015). Because NHEJ is free form the requirement of sister chromatid, this mechanism can be used in cells throughout the cell cycle (Aniukwu et al. 2008; Bennardo et al. 2008; Bartlett et al. 2016). However, the repair accuracy of NHEJ is much less than HR, and NHEJ usually incorporates insertions and deletions (Zaunbrecher et al. 2008). The NHEJ required factor includes Ku70/80 heterodimer (Ku), the DNA-dependent protein kinase catalytic subunit (DNA-PKcs), the X-ray repair cross-complementing 4 (XRCC4)-ligase IV complex, and the XRCC4-like factor protein (XLF, also as known as Cernunnos) (Gerelchuluun et al. 2015). Briefly, NHEJ is initiated with ring-shaped Ku binding that will recruit DNA-PKcs, XLF, and XRCC4/ligase IV. Although the mechanism and the factors are well established, how does these factors assemble and the order for the recruitment of these factors are still unknown. One of well-known mechanism of NHEJ assembly is that XRCC4 and XLF can form long filaments to twine on the DNA (Hammel et al. 2010). This filament can tether and approximate DSB ends to be further polymerized and ligated (Mahaney et al. 2013).

PAXX was identified in 2015 and participates in NHEJ via interaction of Ku80 (Ochi et al. 2015; Xing et al. 2015). PAXX promotes NHEJ in vitro in a Ku80-dependent manner and cells with PAXX deficiency showed increased sensitivity to ionizing radiation, which cause DNA damages that are majorly repaired by NHEJ. Therefore, PAXX is an important new component participates in NHEJ. Xing et al. observed that PAXX and XLF function in both common and parallel pathways (Xing et al. 2015). Interestingly, like XLF-deficient mice, PAXX-deficient mice are viable but with slight increase of ionizing radiation but no impaired immune phenotypes, which are hallmarks of NHEJ deficiency. Jackson and his colleagues further found that PAXX and XLF generate synthetic lethality in mice (Balmus et al. 2016). PAXX and XLF double knockout mice showed embryonic lethality combined with similar phenotypes generated by XRCC4 and ligase IV deficiency. Redundancy of PAXX and synthetic lethality generated by PAXX and XLF have been repeated by several groups (Kumar et al. 2016; Lescale et al. 2016; Tadi et al. 2016; Hung et al. 2017; Gago-Fuentes et al. 2018). Liu et al. found that PAXX accumulates Ku in vivo at DSB ends suggesting that PAXX might participate in NHEJ at early stage (Liu et al. 2017). Since PAXX is redundant in NHEJ and can play a role independent of XLF, we hypothesize that PAXX may also function in other DNA repair pathways, such as homologous recombination or base excision repair (BER).

Glioma is the most common primary cancer in the central nervous system and detected in 6.6 per 100,000 individuals in the USA (Ostrom et al. 2017; Persaud-Sharma et al. 2017). Unfortunately, almost 50% of glioma patients developed glioblastoma, which is the most aggressive glioma form with around 1 year survival duration after diagnosis. Conventional therapies of glioblastoma include surgery, radiotherapy, and chemotherapy. TMZ is the most commonly used chemotherapy for glioblastoma. However, lack of significant improvement of patient survival, developing drug resistance in recurrent tumor, and short of alternative chemotherapy drugs are major obstacles for TMZ in glioma treatment. Therefore, generating new and potent small molecule targeting proteins that are responsible for TMZ resistance could be a promising strategy to overcome TMZ resistance and prolong patient survival. Our study found that PAXX deficiency improved TMZ potency in TMZ resistance glioma cells and this is likely due to participation of PAXX in a variety of DNA repair pathways.

## Materials and Methods

### Generate PAXX-Deficient U87 Cell Line by Using CRISPR/Cas9

In this study, the PAXX-deficient U87 cell was established by using CRISPR/Cas9 according the previous study (Chu et al. 2015) with a few modifications. Briefly, Cas9 along with PAXX guide RNA plasmid was constructed by ligating oligonucleotide duplexes, which targets exon1 of PAXX, into BbsI cut pX330-U6-Chimeric_BB-CBh-hSpCas9 (Addgene, Cat. No. 42230, a gift from Feng Zhang). The plasmid was transfected into U87 cell line along with pcDNA3.1.puro by lipofectamine 2000 and incubated for 72 h. Successfully transfected cells were selected by media containing 100 μg/ml puromycin for 48 h. Cells were harvested and seeded in 96-well plate at concentration of 500 cells/ml and incubated for 2–3 weeks. Induvial clones were passaged, expanded, and screened for PAXX expression by using Western blot assay.

### Cell Lines and Reagents

U87 cells (ATCC, HTB-14) and PAXX-deficient derivatives were cultured at 37 °C in 5% CO_2_ atmosphere in EMEM with 10% fetal bovine serum (FBS) for less than 6 months. U87-TMZ-resistant cells were obtained by treating U87 WT cells with 10 to 150 μM TMZ for 3 months. Human PAXX cDNA was synthesized and purchased from Life Technologies. PAXX cDNA was then cloned in Pet28a vector (EMD Millipore, Cat. No. 69864) to generate his-PAXX plasmid. His-PAXX plasmid was transfected by using Lipofectamine 3000 (ThermoFisher Scientific, Cat. No. 3000008) according to the manufacturer’s instruction. Si-XLF and si-pol β (Sigma) were transfected into U87 cells by using Lipofectamine RNAiMAX (ThermoFisher Scientific, Cat. No. 13778075). GST-tagged human pol β was cloned from pIRESpuro-Flag-pol b (addgene, Cat. No. 23257) into pET-GST vector (addgene, Cat. No. 42049).

### HR and NHEJ Reporter Assay

Both reporters were previously reported (Seluanov et al. 2010). Briefly, 20 μg of NHEJ or HR reporter plasmid was linearized by 50 U of NheI in 50 μl for 6 h at 37 °C. Linearized DNA were then purified by using Qiagen gel extraction kit (Qiagen, Cat. No. 28704). 0.5 μg of linearized DNA was transfected into U87 cells by using lipofectamine 3000 following manufacturer’s recommendations. Cells with chromosomally integrated reporter constructs were selected by incubating in media with 1 mg/ml geneticin 24 h after transfection for 2 weeks. Plasmid integrated cells were seeded at 3 × 10^5^ cells/ml in a 6-well plate and cultured for 24 h to allow adhere. 2 μg/well of I-SceI was transfected into the cell by lipofectamine 3000 and incubated for 48 h. Cells were harvested by using trypsin and re-suspended in PBS by pipetting 10 times. GFP-positive cells were count by flow cytometry (Beckman Coulter).

### Western Blot Analysis

The potential proteins were detected using Western blot analysis as previously described (Wang et al. 2016; Pu et al. 2016). Briefly, protein concentrations were measured using Bradford methods, and then the protein samples were re-suspended in SDS-PAGE sample buffer, and boiled for 5 min at 95 °C. The samples were loaded and separated on a 12% polyacrylamide gel (29:1) at 120 V for 1.5 h on electrophoresis apparatus (Bio-Rad). Separated samples were transferred to nitrocellulose membrane at 100 V and 4 °C in cold room for 45 min. Membrane was blocked by 3% BSA dilute in PBS with 0.1% Tween20 and probed by relevant antibody followed by HRP-conjugated rabbit secondary antibody. The protein signal was developed by SuperSignal™ west Femto Maximum Sensitivity Substrate (ThermoFisher Scientific, Cat. No. 34096) and detected by ChemiDoc™ (Bio-Rad). Antibodies: anti-PAXX (abcam, Cat. No. 126353), anti-pol β (abcam, Cat. No. 26343), anti-actin (abcam, Cat.No. 8227).

### Cell Viability Assay

Cells were seed at 4 × 10^3^ cells/well and cultured for overnight. Cells were incubated with drug for 72 h and the cell viability was detected by using sulforhodamine B (SRB) assay. Cells were fixed by 100 μl/well of 10% trichloroacetic acid at 4 °C for 1 h. Plate was washed four times with slow running tap water and air-dried for 1 h at room temperature (RT) or 20 min in fume hood. Cells were stained by 100 μl/well 0.02% SRB in 1% acetate acid for 1 h at RT. Plates were washed for three times with 200 μl/well 1% acetate acid and air-dried. 200 μl/well of 10 mM Tris-HCl, pH 10.5 was added in each well to extract SRB with 1 h shaking on an orbital shaker. The absorbance was measured at 510 nm by microplate reader (Bio-Tek).

#### Ionizing Radiation and Clonogenic Survival Assay

Cells were re-suspended in 15 ml cell culture and exposed to ionizing radiation by Gammator 50 ^137^Cs source irradiator. Exposed cells were seeded at 500 cells/well in 6-well plate and incubated for 2 weeks or till colonies are visible. Colonies were stained by crystal violet solution for 20 min at room temperature. Plate was washed with slow running tap water till colonies are visible and the background was clean. Colonies were counted by using Quantity One 1-D analysis software.

### Protein Expression and Purification

Bacteria expressed PAXX and PARP1 proteins were collected and purified as previously described Sun et al. (2015, b). Briefly, BL21 (DE3) *E. coli* cells (NEB) were transformed by heat shot method at 42 °C with the plasmid, recovered at 37 °C, and expanded in Luria broth (LB) at 37 °C until the culture reached OD-600 1.0.1 mM isopropyl b-D-thiogalactopyranoside (IPTG) was added to induce protein expression at 18 °C for 16. Induced *E. coli* were harvested by centrifugation at 6000×*g*, 4 °C for 30 min. The medium was removed and the cell pellet was re-suspended in lysis buffer (1× protease inhibitor cocktail, 10 mM EDTA, 20 mM Tris pH 7.9, 600 mM NaCl, 2 mM DTT, 2 mg/ml lysozyme). Lysate was then sonicated by using Branson Sonifier 15%, 10× 10 s to shear DNA to lower viscosity. The clarified supernatant was collected by centrifugation for 15 min at 20,000×*g* at 4 °C. Tagged protein was bound to 0.5 ml of glutathione beads (GST-tagged protein) or Ni-NTA (Qiagen) (his-tagged protein) in 50 ml tube with mixing at 4 °C for 2 h. The beads were packed onto a column and washed with wash buffer (1× protease inhibitor cocktail, 10 mM EDTA, 250 mMNaCl, and 0.5 mM DTT). The his-tagged protein was eluted from the beads by using elution buffer (5 mM imidazole in wash buffer with 5% glycerol). The GST-tagged protein was eluted from the beads by using elution buffer (10 mM glutathione in wash buffer with 5% glycerol). Protein was concentrated by using Pierce™ Protein Concentrator PES, 3 K MWCO (ThermoFisher, Cat. No. 88514) and 30 K (ThermoFisher, Cat. No. 88529) for PAXX and PARP1 respectively in dialyzing buffer (50 mM Tris pH 7.9, 50 mM NaCl, 1 mM EDTA, 2 mM DTT, and 10% glycerol) to lower the salt concentration. Purified protein was frozen using liquid nitrogen and stored at − 80 °C. Protein concentrations were determined by Bradford assay.

### Affinity Capture Assay

Fifty micrograms of his-PAXX was bind to 50 μl of Ni-NTA beads (50% slurry) in 400 μl binding buffer (25 mMTris pH 7.9, 150 mMNaCl, 1 mM EDTA, 1 mM DTT) at 4 °C with gentle mix for 1 h. The resin was collected by centrifugation at 4 °C, 1000×*g* for 1 min followed with washing with 0.5 ml of binding buffer for three times. The resin was dried by centrifugation at 4 °C, 1000×*g* for 1 min and incubated with 50 μg of GST-tagged protein in a final volume of 500 μl at 4 °C with gentle mix for 2 h. The resin was washed with binding buffer for three times and the supernatant was removed by centrifugation at 1000×*g* for 1 min. Bound protein was eluted from the resin by incubating the resin with 50 μl of wash buffer with 5 mM imidazole for 15 min at 4 °C. The mix was centrifuged at 1000×*g* for 1 min and the supernatant was collected. SDS-PAGE loading buffer was added to the sample and boiled for 5 min. The sample then loaded and separated on a SDS-PAGE gel and transferred to nitrocellulose membrane. The GST-tagged protein is observed by using Western blot.

### Statistical Analysis

All data were expressed as mean ± SD and the statistical analysis was performed using SPSS 18.0 (Chicago, IL). Differences between groups were analyzed using one-way analysis of variance (ANOVA). Two-sided *p* values less than 0.05 were considered statistically significant.

## Results

### Generation of PAXX-Deficient Glioma Cell Line by Using CRISPR/Cas9

**Fig. 1:**
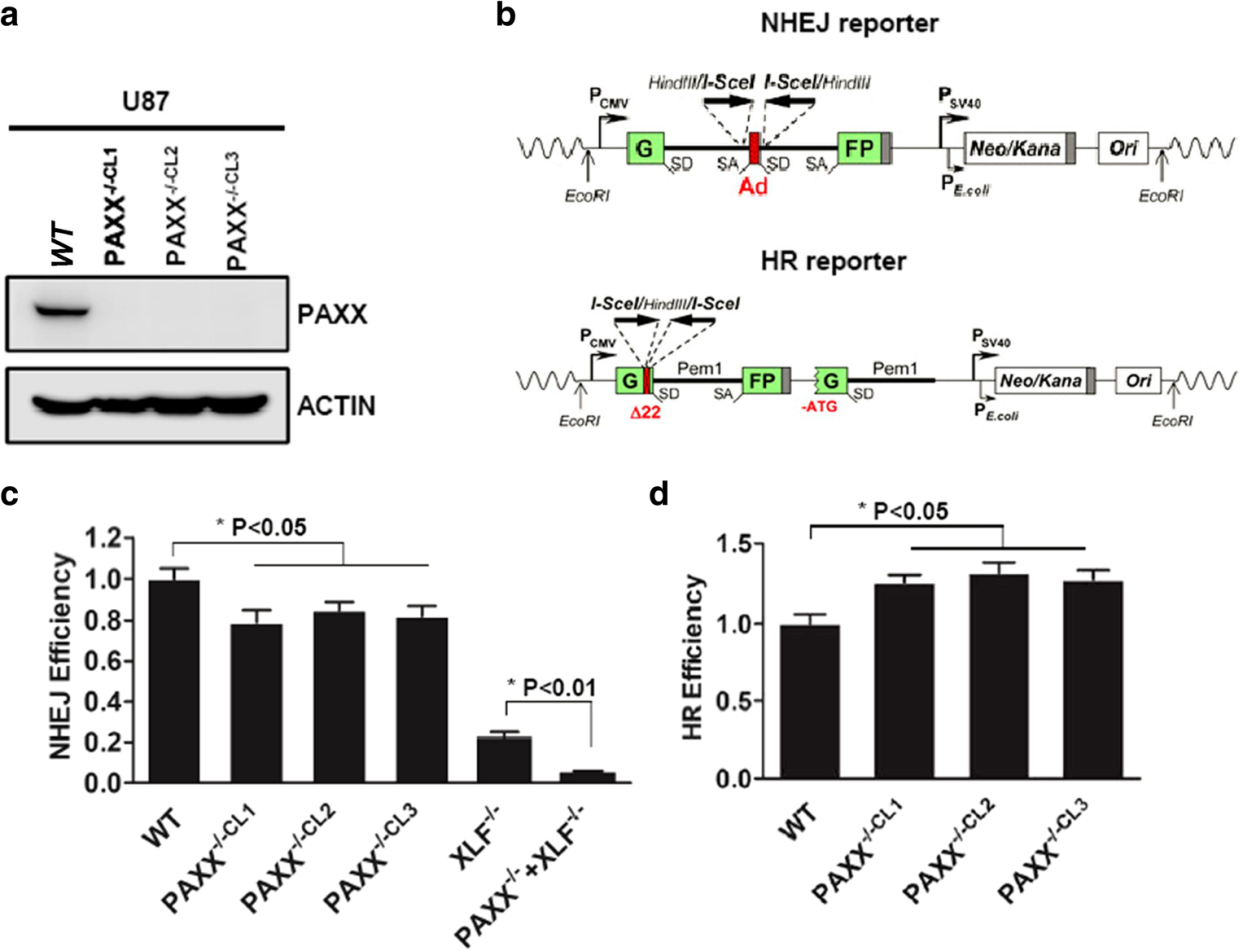
Generation of PAXX-deficient glioma cell line by using CRISPR/Cas9. **a** Western blot of endogenous PAXX expression in U87 WT cell line and three clones of U87PAXX-deficient cell lines (PAXX-/-CL1, PAXX-/-CL2, and PAXX-/-CL3) generated by CRISPR/Cas9. **b** Non-homologous end joining (NHEJ) and homologous recombination (HR) reporter assay diagram. Adapted from Seluanov 2010. Reporter plasmid contains GFP gene that is separated byPem1 intron. DSBs are generated by introducing I-SceI. **c** Quantification of GFP events generated by NHEJ and **d** HR in U87 WT and three U87 PAXX-deficient cell lines. The GFP events were normalized to that in U87 WT cells. Each result represents three independent experiments

To determine whether PAXX participates in DNA repair pathways in glioma cells, we first used CRISPR/Cas 9 method to generate PAXX-deficient cell lines. The guide RNA was designed by the “crispr.mit.edu” on line tool to target exon1 of PAXX. Knockout was achieved by introducing premature stop codon in PAXX coding gene. We harvested 23 knockout candidate clones and picked three clones based on the Western blot result against PAXX antibody. All three PAXX knockout clones showed PAXX protein deficiency as compared to wild-type (WT) U87 cells (Fig. 1a).

Because PAXX participates in NHEJ and its role was well established. We first used NHEJ reporter assay to further verify the PAXX deficiency in U87 cells. The NHEJ reporter is a plasmid contains GFP gene, which is interrupted by pem1 gene flanked by I-SceI endo-nuclease recognition sites (Seluanov et al. 2010). Since these I-SceI sites are located in an invert order, the DSB generated by I-SceI are incompatible, which best mimic the natural DSB generated in cells (Fig. 1b). Once NHEJ repair performs, GFP gene will be repaired and GFP signal can be detected to indicate NHEJ efficiency. We observed that, as expected, PAXX-deficient U87 cells showed slight decrease of NHEJ (Fig. 1c). Consistent with other group’s result, PAXX and XLF deficiency generates synergistic effect on NHEJ efficiency in cells (Fig. 1c). We are also interested in whether PAXX participates in HR in glioma cells because HR can compete with NHEJ for and is usually upregulated when NHEJ is impaired (Pierce et al. 2001). We then used HR reporter assay in U87 cells with PAXX deficiency. Similar to NHEJ reporter, HR reporter is also a plasmid-based assay that the repair of DSB generated by I-SceI can generate GFP signal. Importantly, this HR ensures GFP is resulted from templated dependent repair instead of direct end joining because the GFP gene contains a 22-nt deletion that cannot be fixed by NHEJ. We observed that PAXX deficiency indeed increased HR efficiency by 18–21% (Fig. 1d). Therefore, our data strongly demonstrated that we successfully generated PAXX-deficient U87 cells.

### TMZ Induced PAXX Expression and PAXX Is Upregulated in TMZ-Resistant U87 Cells

**Fig. 2:**
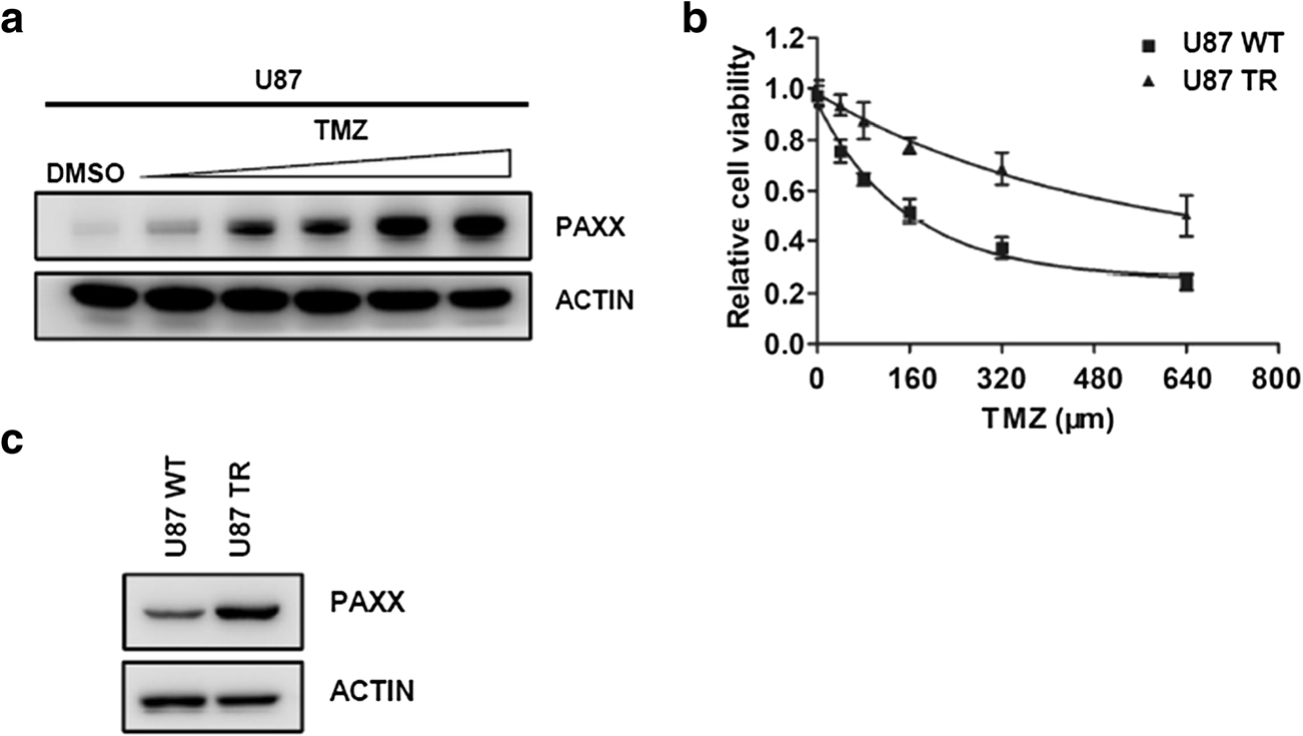
TMZ induces PAXX expression and PAXX is upregulated in TMZ-resistant U87 cells. **a** Western blot of PAXX expression in U87 WT cells treated with TMZ. TMZ concentrations: 0, 20, 40, 80, 160, and 320 μM. **b** U87 TMZ-resistant cell line (U87 TR) is generated. TMZ concentrations: 0, 40, 80, 16, 320, and 640 μM. **c** Western blot of PAXX expression in U87 WT and U87 TR cell lines

DNA repair is one of the most important reasons for TMZ drug resistance (Kondo et al. 2011; Castro et al. 2015). Since PAXX participates in HR and NHEJ, which are the two major DNA double-strand repair pathway in mammalian cells, we hypothesize that PAXX contributes to TMZ resistance. We observed that TMZ induced PAXX expression in U87 cells after 3 days (Fig. 2a). In order to evaluate PAXX protein level in long-term TMZ-treated cells, we generated TMZ-resistant U87 cell line (U87 TR) by incubating U87 WT cells with TMZ for 3 months. The U87 TR cell line showed significant resistance as compared to WT cells by using SRB cell viability assay (Fig. 2b). Interestingly, we observed that PAXX expression increased in TMZ-resistant cell line (Fig. 2c). These data suggest that PAXX contributes to TMZ resistance in glioma cell line.

### PAXX Interacted with Pol βand Improved BER Efficiency

**Fig. 3:**
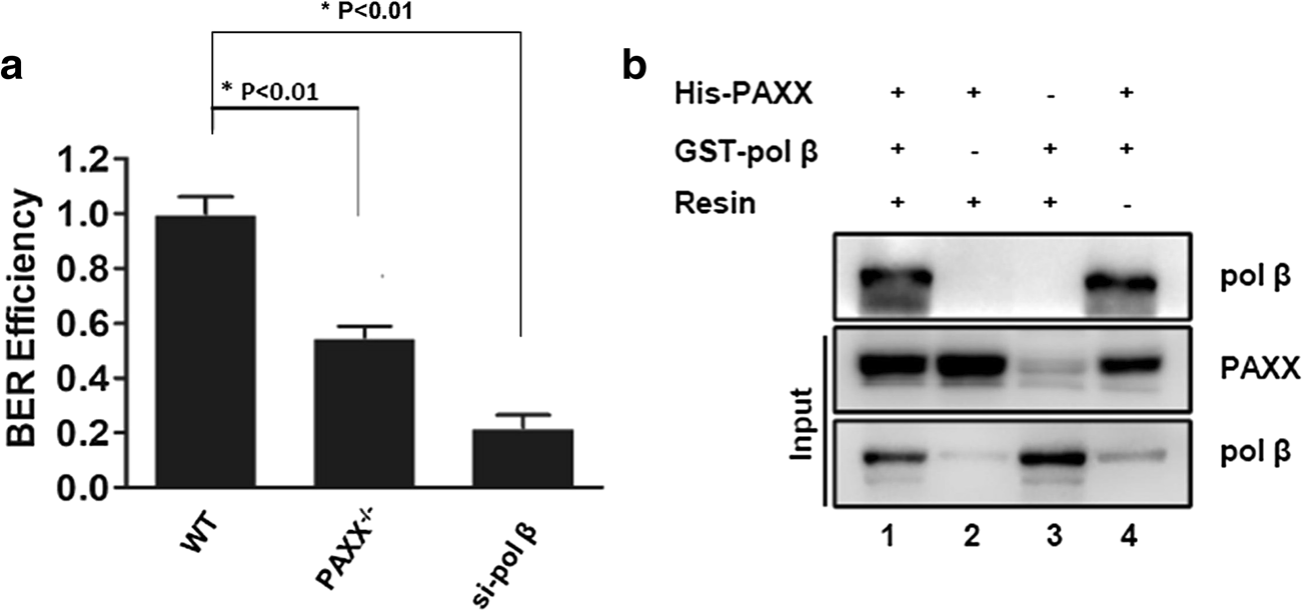
PAXX interacts with pol β and improves BER efficiency. **a** Quantification of BER reporter assay on U87 WT, PAXX-deficient and pol β knockdown cell lines. **b** Western blot of affinity capture assay. His-PAXX was used to capture GST-pol β on Ni-NTA resin. Lane 4: 1 ng of purified protein as positive controls. Samples were separated by SDSPAGE, Western transferred and pol β was detected by using anti-pol β antibody

TMZ is a pro-drug that can alkylate DNA, specifically, adding a methyl group to purine base (Bady et al. 2018). TMZ generates cancer cell death majorly by introducing O6-methylguanine (O6-MeG) to induce cell cycle arrest at G2/M and to further result in apoptosis (Zhang et al. 2012). Repair of O6-MeG can be achieved by directly removal by methylguanine methyltransferase (MGMT) or tolerated by mismatch repair. Therefore, MGMT or lack of mismatch repair can contribute to TMZ resistance (Naumann et al. 2009; Perazzoli et al. 2015). Interestingly, more than 80% of DNA damage generated by TMZ are *N*-methylated bases that are substrates for BER pathway. Therefore, TMZ resistance may also partially, yet importantly, caused by BER (Sobol and Wilson 2001; Tentori and Graziani 2002). We then evaluated BER efficiency by using a GFP-based reporter assay in PAXX-deficient cells and found that U87 cells lack PAXX displayed 50% less BER efficiency (Raetz et al. 2012) (Fig. 3a). While deficiency of polymerase beta (pol β), which is an essential factor in BER, generates much more significant BER depletion (Sobol and Wilson 2001; Cabelof et al. 2003).

To understand how PAXX participates in BER, we incorporated affinity capture assay to detect protein-protein interactions between with purified his-tagged PAXX (his-PAXX) and GST-tagged BER factors. We observed that PAXX interacted with pol β, which participates in later stage of BER (Fig. 3b), in vitro. To eliminate DNA contaminations, which may result in false positive result, we used DNA nuclease during the reaction for protein-protein interaction. Our result indicates that PAXX participates in BER in glioma cells via interaction with pol β.

### PAXX Depletion Sensitized Glioma Cells to TMZ

**Fig. 4:**
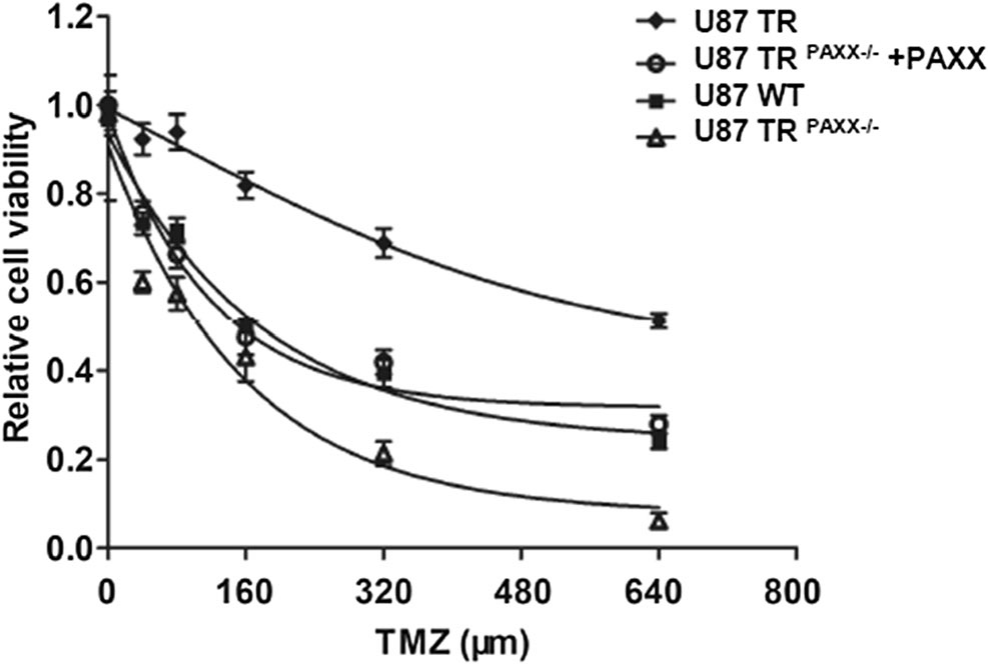
PAXX depletion sensitizes glioma cells to TMZ Cell Survival assay of U87 WT, U87 TR, U87 TR PAXX-/- and U87 TR PAXX-/- +PAXX. TMZ concentration: 0, 40, 80, 160, 320, and 640 μM

Since variety of groups found that the mechanism of TMZ resistance is correlated with DNA repair. Importantly, BER can repair more than 80% of DNA lesions generated by TMZ. Therefore, we hypothesize that PAXX, which contributes to BER, as well as NHEJ efficiency, promotes TMZ resistance in glioma cells. We performed cell survival assay to evaluate TMZ cytotoxicity in PAXX-depleted U87 cells. To this end, we observed that PAXX knockout in TMZ-resistant cells re-sensitized U87 TR cells to TMZ with a 4.3-fold decrease of TMZ IC50 as compared to that in U87 TR cells (Fig. 4). To minimize the off-target effect of PAXX knockout, we expressed PAXX in PAXX-deficient cells and measured the cell viability. We found that ectopic expression of PAXX rescued TMZ resistance in PAXX-deficient cells, indicating that the increased sensitivity to TMZ is indeed caused by loss of PAXX. Our data suggest that PAXX improves BER efficiency and could be a potential target in combinational chemotherapy with TMZ.

## Discussion

PAXX is a new component identified in NHEJ pathway in 2015. It shares conserved present-in-SAS6 (PISA) motif and similar structure with XRCC4 and XLF, therefore, belongs to XRCC4 superfamily (Ochi et al. 2015). Jackson and Xu labs, almost at the same time, revealed the role of PAXX in NHEJ (Xing et al. 2015). To our knowledge, an enzymatic activity of PAXX has not been found yet, indicating PAXX may contribute to DNA repair by serving as a scaffolding protein. It is very interesting to found that PAXX can interact with pol β and promotes BER efficiency. Since PAXX interacts with Ku via its C-terminal domain, we would like to do mutational analysis assay in our following research to determine whether PAXX uses same domain to interact with these two essential factors that participate in separate DNA repair pathways.

DNA accuracy and integrity are essential for cell survival. Therefore, DNA has been targeted in chemotherapy for cancer treatment for decades since the first use of nitrogen mustards. However, cancer cells developed resistance to these DNA damaging drugs very likely caused by upregulated DNA repair. So, DNA repair is believed to be one of the major fields for discovering new drugs that may overcome resistance of DNA damaging agents. TMZ was originally approved by US Food and Drug Administration in 1999 for treatment of refractory adult anaplastic astrocytoma (Middleton et al. 2000) and newly diagnosed glioblastoma since in adult in 2005 (Domingo-Musibay and Galanis 2015). Unfortunately, major portion of glioma patients developed TMZ resistance and left with very limited options for alternative chemotherapeutic drugs. Therefore, it is very important and significant to generate new potent drugs against gliomas or to impair DNA repair to minimize drug resistance.

In conclusion, we found that PAXX not only plays an important role in NHEJ but also contributes to BER. Depletion or inhibition of proteins play multiple roles in several DNA repair pathways, such as PAXX, provides significantly improved efficiency of DNA damaging agents.
